# Cost-effectiveness of artemisinin–naphthoquine versus artemether–lumefantrine for the treatment of uncomplicated malaria in Papua New Guinean children

**DOI:** 10.1186/s12936-017-2081-8

**Published:** 2017-10-30

**Authors:** Brioni R. Moore, Wendy A. Davis, Philip M. Clarke, Leanne J. Robinson, Moses Laman, Timothy M. E. Davis

**Affiliations:** 1 School of Pharmacy, Curtin University of Technology, Perth, WA Australia; 2 School of Medicine and Pharmacology, University of Western Australia, Perth, WA Australia; 3 Melbourne School of Population and Global Health, University of Melbourne, Melbourne, Australia; 4 Papua New Guinea Institute of Medical Research, Madang, Papua New Guinea; 5 Burnet Institute, Parkville, Melbourne, VIC Australia; 6 Division of Population Health and Immunity, Walter and Eliza Hall Institute, Parkville, VIC Australia

**Keywords:** Uncomplicated malaria, Artemether–lumefantrine, Artemisinin–naphthoquine, Children, Cost-effectiveness

## Abstract

**Background:**

A recent randomized trial showed that artemisinin–naphthoquine (AN) was non-inferior to artemether–lumefantrine (AL) for falciparum malaria and superior for vivax malaria in young Papua New Guinean children. The aim of this study was to compare the cost-effectiveness of these two regimens.

**Methods:**

An incremental cost-effectiveness analysis was performed using data from 231 children with *Plasmodium falciparum* and/or *Plasmodium vivax* infections in an open-label, randomized, parallel-group trial. Recruited children were randomized 1:1 to receive once daily AN for 3 days with water or twice daily AL for 3 days given with fat. World Health Organisation (WHO) definitions were used to determine clinical/parasitological outcomes. The cost of transport between the home and clinic, plus direct health-care costs, served as a basis for determining each regimen’s incremental cost per incremental treatment success relative to AL by Day 42 and its cost per life year saved.

**Results:**

In the usual care setting, AN was more effective for the treatment of uncomplicated malaria in children aged 0.5–5.9 years. AL and AN were equally efficacious for the treatment of falciparum malaria, however AN had increased anti-malarial treatment costs per patient of $10.46, compared with AL. AN was the most effective regimen for treatment of vivax malaria, but had increased treatment costs of $14.83 per treatment success compared with AL.

**Conclusions:**

Whilst AN has superior overall efficacy for the treatment of uncomplicated malaria in PNG children, AL was the less costly regimen. An indicative extrapolation estimated the cost per life year saved by using AN instead of AL to treat uncomplicated malaria to be $12,165 for girls and $12,469 for boys (discounted), which means AN may not be cost-effective and affordable for PNG at current cost. However, AN may become acceptable should it become WHO prequalified and/or should donated/subsidized drug supply become available.

## Background

Although malaria remains a major global health issue with nearly half the world’s population still at risk and ongoing transmission in 91 countries, substantial progress has been made towards reducing the burden of the disease [1]. The incidence of malaria declined by 21% between 2000 and 2015 [1], and the proportion of global disability adjusted life years attributable to malaria fell from 3.5 to 2.3% over same period representing a drop in ranking relative to other causes from 7th to 14th [2]. This improvement reflects more effective vector control and case management [1].

Artemisinin-based combination therapy (ACT) is the World Health Organization (WHO) recommended first-line treatment for uncomplicated malaria [1]. Five different artemisinin-based combinations, artemether–lumefantrine (AL), artesunate–amodiaquine, artesunate–mefloquine, dihydroartemisinin–piperaquine (DHA–PQP) and artesunate–sulfadoxine–pyrimethamine, currently meet the stringent WHO regulatory standards for prequalification with the choice of ACT based, at least in part, on local parasite drug resistance [1]. The incorporation of ACTs into malaria control programmes has contributed to the global decline in malaria-related mortality and morbidity, but effective therapy remains a challenge in geo-epidemiological settings where there is transmission of multiple *Plasmodium* species [3]. Papua New Guinea (PNG) is a case in point. In 2011, PNG national malaria treatment guidelines were modified to include AL as first-line treatment for uncomplicated *Plasmodium falciparum* and *Plasmodium vivax* infections [4]. These changes reflected WHO contemporary recommendations and were underpinned by the results of a randomized clinical trial that demonstrated that AL was the most efficacious [5] and cost-effective [6] ACT for falciparum malaria in PNG. Although DHA–PQP was more efficacious in vivax malaria, the cost and complexity of having different first-line treatments for cases of falciparum and vivax malaria, meant that it was included as an alternative second-line regimen [4, 5]. Given that potentially preventable morbidity and mortality due to vivax malaria despite AL therapy remained a concern [7], a search for more broadly effective and affordable formulations of ACT was recommended [5].

Of the few potential alternative ACT, artemisinin–naphthoquine (AN) was one candidate. This ACT was already available in the private sector in PNG as single-dose treatment for uncomplicated malaria despite not being prequalified. Preliminary pharmacokinetic and safety studies in PNG children provided good evidence that the recommended single AN dose given daily for 3 days (a duration recommended by the WHO for all ACT medicines to increase cure rates and reduce the development of parasite resistance [8]) would be safe, well tolerated and efficacious treatment for uncomplicated malaria [9, 10], with the long elimination half-life of naphthoquine (t_1/2_ = 23 days) [10] likely to provide more extended suppression of late post-treatment *P. vivax* emergence than the piperaquine component of DHA–PQP.

A randomized comparative safety and efficacy trial of AL and AN was, therefore, performed in young PNG children with uncomplicated *P. falciparum* and *P. vivax* infections in coastal Madang Province [11]. The results showed that AN was non-inferior to AL for falciparum malaria with the same high cure rate, but that it was superior to AL for vivax malaria. However, the cost of using three times the recommended dose of a non-subsidized ACT is likely to be relatively high [12, 13], which would be an impediment to its uptake in developing countries with similar epidemiology to that in malaria-endemic areas of PNG. The aim of the present study was, therefore, to assess the relative cost-effectiveness of AL and AN for the treatment of uncomplicated malaria in Melanesian children aged 0.5–5.9 years.

## Methods

### Patients

**Fig. 1:**
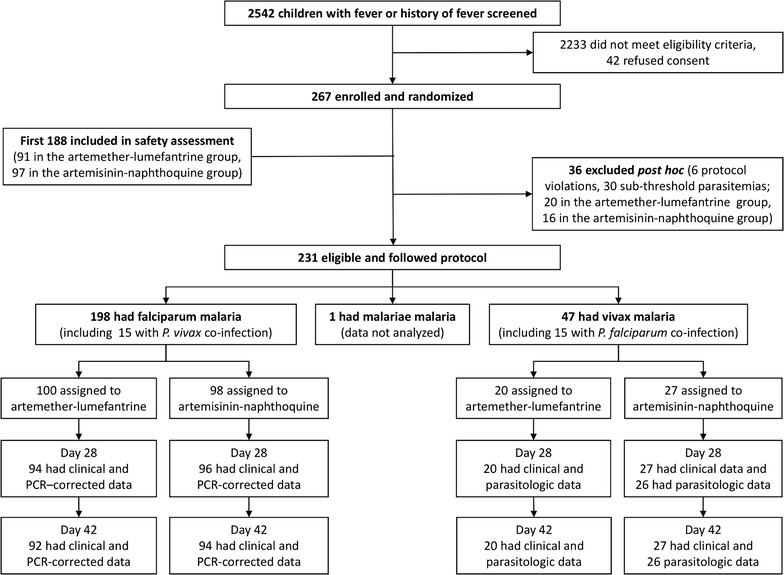
Consort diagram showing numbers of patients from screening to Day 42 assessment. PCR-corrected denotes correction for re-infections identified by PCR genotyping of polymorphic parasite loci (adapted from [11])

Between 28 March 2011 and 22 April 2013, an open-label, randomized, parallel-group trial of AL and AN was conducted at the Mugil and Alexishafen Health Centres (Australian New Zealand Clinical Trials Registry ACTRN12610000913077). The trial was approved by the PNG Institute of Medical Research Review Board, the Medical Research Advisory Committee of PNG, and the University of Western Australia Human Research Ethics Committee. Children aged 0.5–5.9 years presenting with blood slide positive uncomplicated falciparum or vivax malaria were eligible to participate. Full details of study procedures have been published [11]. Using a computer-generated block randomization (24 children per site), participants were allocated 1:1 to either AL (1.7 mg/kg artemether plus 10 mg/kg lumefantrine; Novartis Pharma, Basel, Switzerland) twice daily for 3 days, or to AN (20 mg/kg artemisinin plus 8 mg/kg naphthoquine; Kunming Pharmaceutical Corporation (KPC), Yunnan, China) daily for 3 days. As recommended by the manufacturer, AL was administered as 1–3 whole tablets per dose with 250 ml of milk, whilst AN was given as 1–4 whole tablets with water, with the number of tablets given per dose based on manufacturer recommended weight/dose ranges. Children who vomited within 30 min of drug administration were retreated. The present economic evaluation included all children randomized and followed according to the study protocol (198 with falciparum malaria, 47 with vivax malaria; see Fig. 1).

### Clinical and parasitological methods

An initial standardized clinical assessment including measurement of the axillary temperature was performed and blood was drawn for blood film microscopy, measurement of haemoglobin (Hb) and blood glucose, hepatic and renal function, and a full blood count [11]. A modified assessment was repeated on Days 1, 2, 3, 7, 14, 28 and 42. All blood films were examined by two independent microscopists with parasite density calculated from the number of parasites per 200–500 leucocytes and an assumed leucocyte count of 8000/µl.

Efficacy was assessed using WHO definitions [14], specifically (i) early treatment failure (ETF) or the development of signs of severity or an inadequate parasitological response by Day 3, (ii) late parasitological failure (LPF) or the development of parasitaemia between Days 4 and 42, (iii) late clinical failure (LCF) if the LPF was accompanied by fever, or (iv) adequate parasitological and clinical response (ACPR) otherwise. These outcomes were corrected for re-infection by polymerase chain reaction (PCR) genotyping of the falciparum malaria cases, while *P. vivax* recrudescence was determined by genotyping [11]. No children developed severe malaria during follow-up. Two LCFs were identified at the Day 42 assessment and received AL treatment as per protocol and PNG national treatment guidelines.

### Economic analyses

The perspective of the present analyses was societal. Direct health care costs (AL and AN treatment costs, visits to health centres and tests, and a course of rescue antimalarial therapy when required) plus travel costs were estimated. An incremental cost-effectiveness analysis was performed in which the net costs and net effectiveness of AN were compared with those of conventional AL treatment and expressed as ratios. All analyses and comparisons were performed on both a per protocol (PP) and modified intention to treat (mITT) basis. The PP analyses included children with complete follow-up or confirmed treatment failure, and excluded those treated for malaria without confirmatory microscopy or who defaulted from follow-up. These excluded patients were retained in the mITT which utilized i) a worst-case approach (ETF assumed for Day 3 exclusions, LPF/LCF otherwise) and ii) a best-case approach (all missing follow-up blood films assumed parasite-negative). In a secondary analysis, we extrapolated outcomes to estimate the increase in life expectancy of the most effective treatment based on estimated mortality associated with *P. falciparum* and remaining life expectancy.

For each patient, standardized data were collected at each scheduled clinic visit and at extra, unscheduled “sick day” visits. These included doses of all drugs used for treating malaria, its symptoms and its complications (trial medication, rescue AL, paracetamol, iron and folate supplements). Unit costs were obtained from the PNG National Department of Health [15], Interpath Services Pty Ltd (Heidelberg West, Victoria, Australia), HemoCue Australia Pty Ltd (Tumbi Umbi, New South Wales, Australia), Access Bio Inc. (Somerset, NJ, USA), participating clinics and local suppliers (see Table 1), and were combined with resource volumes to obtain a net cost per patient during follow-up. Mean net costs and associated 95% confidence intervals (CI) were calculated for each treatment arm. Costs are reported undiscounted due to the relative brevity of the trial, and in 2012 US$ values using the average exchange rate during 2012 of 1 PNG Kina (PGK) = US$0.490757 [16].

**Table 1:** Main unit costs and sources for the two treatment arms

Item	Unit cost (2012 PGK)	Unit cost (2012 US$)	Source	Comments
Health clinic visit				
Outpatient	3.00	1.472	Alexishafen/Mugil Health Centres	Includes examination by nurse and treatment
Inpatient	10.00	4.908	Alexishafen/Mugil Health Centres	All treatment costs
Transport cost to Alexishafen Health Centre				
PMV (return)	4.00	1.963	Alexishafen Health Centre	For average distance (PGK2 close—PGK6 far)
Hire vehicle (night transport after PMV hours)	35.00	17.176	Alexishafen Health Centre	Range PGK20-PGK50
Ambulance (emergency)	30.00	14.723	Alexishafen Health Centre	
Transport cost to Mugil Health Centre				
PMV (return)	6.00	2.945	Mugil Health Centre	For average distance (PGK2 close—PGK10 far)
Hire vehicle (night transport after PMV hours)	40.00	19.630	Mugil Health Centre	Range PGK30- PGK50
Ambulance (emergency)	30.00	14.723	Mugil Health Centre	
Malaria treatment				
A + L	0.396	0.194	PNG National Department of Health, 2012	National standard treatment
A + N	3.75	1.840	Local pharmacy over-the-counter	
Other medications				
Paracetamol syrup 120 mg/5 ml	0.010/ml	0.005/ml	PNG National Department of Health, 2012	15 mg/kg given if axillary temperature > 37.5 °C
Paracetamol tablets, 500 mg	0.011	0.005	PNG National Department of Health, 2012	15 mg/kg given if axillary temperature > 37.5 °C
Paracetamol suppository, 250 mg	0.226	0.111	PNG National Department of Health, 2012	15 mg/kg given if axillary temperature > 37.5 °C
Albendazole 200 mg tablet	0.025	0.012	PNG National Department of Health, 2012	For hookworm on Day 42 if Hb < 90 g/l
Fefol (FeSO_4_ 200 mg, folic acid 0.4 mg)	0.027	0.013	PNG National Department of Health, 2012	Day 42 if Hb < 90 g/l
Amodiaquine 100 mg tablet	0.024	0.012	PNG National Department of Health, 2012	Day 42 if Hb < 90 g/l and spleen grade ≥ 3
Tests				
Rapid diagnostic test	2.241	1.100	Access Bio Inc.	CareStart Malaria HRP2/pLDH (Pf/PAN) Combo; Baseline
HemoCue Hb point of care (POC) test	3.454	1.695	HemoCue Australia Pty Ltd	
HemoCue glucose POC test	3.896	1.912	HemoCue Australia Pty Ltd	
Malaria microscopy, blood slide and reading	3.580	1.757	Interpath, national salary	Days 0–42; includes price of blood slide, stain, 15 min salary to read slide
Other				
Milk (250 ml tetra packs full cream)	1.708	0.838	K41.00/24 packs wholesale (2011)	
Syringe disposable 10 ml with 21-G needle	0.210	0.103	PNG National Department of Health, 2012	
Gloves, disposable	0.136	0.067	PNG National Department of Health, 2012	PGK6.82/box non-sterile gloves
Blood lancet, disposable	0.032	0.016	PNG National Department of Health, 2012	PGK6.38/box of 200 lancets
Alcohol wipe × 1 each blood sample	0.017	0.008	PNG National Department of Health, 2012	PGK1.74/box of 100 sterile wipes
Cotton wool swab × 1 each blood sample	0.007	0.003	PNG National Department of Health, 2012	PGK3.55/500 g pack

Per tablet or test except where indicated otherwise

All study participants were scheduled to attend eight times including Day 0 but excluding sick days. However, the frequency and type of visits are different in a usual care (non-trial) situation. Therefore, a complementary analysis, in which costs of AL or AN treatment are based on a single clinic visit at which malaria is diagnosed and treated, was conducted. The same number of subsequent sick day visits was assumed except for ETFs where the scheduled Day 1–3 clinic visit was replaced by a sick day visit. Each patient’s actual trial visit costs were replaced by the estimated standard practice cost depending on allocation. It was assumed that no-cost forms of fat (from a normal diet or breastfeeding in the case of young infants) were consumed with AL.

The primary endpoint of the trial was treatment failure by Day 42 [5]. A secondary analysis of the lifetime benefits of using AN for *P. falciparum* was performed by extrapolating the benefits using lifetables to estimate the potential life years saved. All comparisons were carried out separately for *P*. *falciparum* and *P*. *vivax* on both a PP and a mITT basis. All results are reported as means and SDs or mean differences and 95% CIs. The CIs for the key incremental cost-effectiveness ratios (ICERs) were estimated using the bootstrap approach with 1000 repeated random samples drawn with replacement from the original data. Bootstrap confidence intervals were constructed with the bias-corrected percentile method [17]. The effect of assumptions on main results was examined by sensitivity analyses involving undertaking a cost-effectiveness analysis using best and worst case mITT assumptions. Data were analysed using IBM SPSS Statistics 22 (IBM Corporation, Armonk, New York, USA).

## Results

The 186 children in the PP *P. falciparum* analysis were of mean ± SD age 3.7 ± 1.3 (range 0.8–5.9) years and 53.8% were boys, while 41.3% of the 46 children in the PP *P. vivax* analysis were boys and the mean age was 3.1 ± 1.2 (range 0.5–5.8) years.

### Costs

Table 2 shows the mean cost per patient and the mean cost difference between AL and AN over the duration of the study by category of cost and allocation for *P. falciparum* and *P. vivax* in both trial and usual care settings. For *P. falciparum*, AN had increased anti-malarial treatment costs for each patient of $10.46 (95% CI $9.77–$11.16) on average compared with AL. There were no significant differences between AL and AN in other costs. Total usual care costs were significantly higher in the AN group compared with the AL group.

**Table 2:** Per protocol analysis showing mean ± SD per patient costs and mean (95% CI) cost differences in 2012 US$ for AL vs. AN given for falciparum (upper panel) or vivax (lower panel) malaria in usual care and trial settings

	AL	AN	AN vs. AL
Falciparum malaria			
Number	92	94	
Anti-malarial treatment	1.58 ± 0.56	12.04 ± 3.35	10.46 (9.77 to 11.16)*
Paracetamol	0.003 ± 0.002	0.003 ± 0.003	0.000 (− 0.001 to 0.001)
Clinic visits	5.53 ± 1.63	5.23 ± 0.77	− 0.29 (− 0.66 to 0.08)
Total (usual care)	7.66 ± 2.29	17.28 ± 3.40	9.61 (8.78 to 10.45)*
Excess costs for trial	54.71 ± 1.31	49.56 ± 1.54	− 5.15 (− 5.56 to − 4.74)*
Total (trial)	62.37 ± 3.21	66.84 ± 3.83	4.46 (3.44 to 5.49)*
Vivax malaria			
Number	20	26	
Anti-malarial treatment	1.41 ± 0.48	12.46 ± 2.72	11.05 (9.93 to 12.16)*
Paracetamol	0.003 ± 0.003	0.002 ± 0.002	− 0.000 (− 0.002 to 0.001)
Clinic visits	5.12 ± 0.00	5.32 ± 1.04	0.20 (− 0.27 to 0.67)
Total (usual care)	7.41 ± 1.16	17.78 ± 3.20	10.38 (9.00 to 11.76)*
Excess costs for trial	54.72 ± 0.13	49.76 ± 0.43	− 4.96 (− 5.16 to − 4.76)*
Total (trial)	62.12 ± 1.14	67.54 ± 3.45	5.42 (3.95 to 6.89)*

* *P* < 0.001 vs. AL; negative cost differences indicate cost-savings associated with AN

For *P. vivax* infections, AN increased anti-malarial treatment costs for each patient by an average of $11.05 (95% CI $9.93–$12.16) compared with AL (see Table 2). There were no significant differences between AL and AN therapies in other costs. Total usual care treatment costs were significantly higher in the AN group compared with AL treatment.

### Outcomes

In the PP *P. falciparum* analysis of the 186 children who completed the trial, 184 (98.9%) had an ACPR with the highest rate in the AN group (100%) compared with 97.8% in the AL group [11]. For the 32 children who attended on Day 42, the rate of ACPR for *P. vivax* was 100% in the AN group compared with 30.0% in the AL group [11]. Table 3 documents the proportion of treatment successes and the costs for each malaria species and each type of analysis (PP and mITT) for a usual care setting. The incremental number of successes and costs together with the ICER are also shown for AN compared with AL.

**Table 3:** Cost-effectiveness analyses by malaria species and type of analysis for a usual care setting

	AL	AN
*Plasmodium falciparum*		
Per protocol (total = 186)		
Number	92	94
Successes (proportion)	0.978	1.00
Mean cost (US$)/patient	7.66	17.28
Incremental successes	–	0.022
Incremental costs (US$)	–	9.61
Incremental cost-effectiveness ratio	–	436.82
Modified intention to treat (N = 198)		
Number	100	98
(i) Worst case		
Successes (proportion)	0.900	0.959
Mean cost (US$)/patient	7.55	17.16
Incremental successes	–	0.059
Incremental costs (US$)	–	9.60
Incremental cost-effectiveness ratio	–	162.71
(ii) Best case		
Successes (proportion)	0.980	1.000
Mean cost (US$)/patient	7.55	17.16
Incremental successes	–	0.020
Incremental costs (US$)	–	9.60
Incremental cost-effectiveness ratio	–	480.00
*Plasmodium vivax*		
Per protocol (total = 46)		
Number	20	26
Successes (proportion)	0.300	1.000
Mean cost (US$)/patient	7.41	17.78
Incremental successes	–	0.700
Incremental costs (US$)	–	10.38
Incremental cost-effectiveness ratio	–	14.83
Modified intention to treat (N = 47)		
Number	20	27
(i) Worst case		
Successes (proportion)	0.300	0.963
Mean cost (US$)/patient	7.41	17.52
Incremental successes	–	0.663
Incremental costs (US$)	–	10.11
Incremental cost-effectiveness ratio	–	15.25
(ii) Best case		
Successes (proportion)	0.300	1.000
Mean cost (US$)/patient	7.41	17.52
Incremental successes	–	0.700
Incremental costs (US$)	–	10.11
Incremental cost-effectiveness ratio	–	14.29

Incremental successes and costs are for AN vs. AL

### Cost-effectiveness

**Fig. 2:**
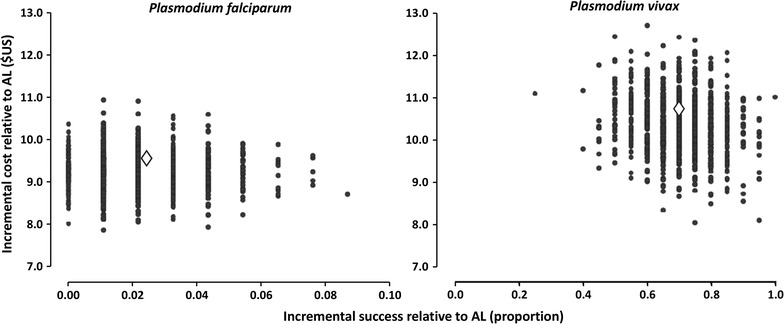
Scatterplots of estimated joint density of incremental costs and incremental effects (treatment successes) by bootstrap re-sampling of AN relative to AL for children with *Plasmodium falciparum* malaria (left) and *Plasmodium vivax* malaria (right) in a usual care setting in Papua New Guinea 2011–2013. The ◊ represents the point estimate for incremental cost and incremental effect (treatment success)

The primary measure of cost-effectiveness is the incremental cost per incremental treatment success relative to the comparator AL. In a usual care setting, AN was more effective but more costly than AL (see scatterplot of estimated joint density of incremental costs and incremental effects of AN vs. AL obtained by bootstrap resampling in Fig. 2). For *P. falciparum*, the average excess cost per treatment success for AN when compared with AL was $436.82 with a 95% bootstrap bias-corrected confidence interval of $200.23–$941.29 and a 68.3% chance that the incremental cost-effectiveness ratio was below $500 per treatment success. For *P. vivax* (see Fig. 2), AN was more effective than AL, but more costly with an average excess cost per treatment success of $14.83 in a usual care setting when compared with AL with a 95% bootstrap bias-corrected confidence interval of $11.06 to $22.17 and a very high probability (99.7%) that the excess cost per success was < $25.

### Cost per life year saved if AL is replaced by AN

A girl and boy aged between 1 and 4 years old in 2012 in PNG could expect to live another 66.6 and 62.1 years, respectively [18]. In PNG in 2012, there were 150,195 reported confirmed cases of malaria and 381 reported malaria deaths, a mortality rate of 0.25% [19], with both cases and deaths concentrated in younger age groups which have yet to develop immunity. The incremental success of AN over AL was 2.2% (100% vs. 97.8%) in children 0.5–5.9 years old. The number of deaths from *P. falciparum* prevented per 1000 cases when treated with AN instead of AL is thus estimated to be 0.0025 × 1000 × 0.022 = 0.055. The increase in average life expectancy for 1000 cases of *P. falciparum* treated with AN is approximately 0.055 × 66.6 years for girls = 3.66 years (or 1.64 years when discounted at 3%) and 0.055 × 62.1 years for boys = 3.42 years (or 1.60 years when discounted at 3%). The extra cost associated with AN versus AL treatment was $19,590 per 1000 cases treated (PP analysis). Therefore, the cost per life year saved was $19,590/3.66 = $5352.46 (or $12,164.63 when benefits are discounted at 3%) for girls and $19,590/3.42 = $5728.07 (or $12,468.75 when benefits are discounted at 3%) for boys.

## Discussion

This is the first economic analysis of AN for treatment of uncomplicated malaria in an area of intense transmission of multiple *Plasmodium* species. The present data show that, while a 3-day course of AN had superior overall efficacy in uncomplicated paediatric malaria, the recommended first-line AL remained the more cost-effective treatment. In PNG children with falciparum malaria, AN was equally efficacious but far more costly than AL. In the case of vivax malaria, AN was significantly more efficacious than AL but only slightly more costly in the usual care setting. The most significant contributor to the difference in costs between AN and AL is the fact that AL is a subsidized prequalified first-line therapy provided with financial assistance from a Global Fund Affordable Medicines Facility Grant [13], while AN can only be purchased over-the-counter at pharmacy-determined commercial cost. The availability of donated or subsidized drugs and willingness to pay will, therefore, determine the choice of regimen by the consumer outside the public health system.

The only formulation of AN currently available is that manufactured by KPC under the brand name ARCO^®^. It satisfies WHO recommendations for universal combination therapy for uncomplicated malaria but has not yet met the WHO prequalification manufacturing standards [20]. As a result, AN is not eligible for financial subsidy, inclusion on competitive pricing indices, or global distribution by International agencies including UNICEF, the Global Fund and Unitaid [13, 20]. As KPC continues to distribute ARCO^®^ as a single-dose treatment, a regimen which is inconsistent with WHO recommendations for 3 days of ACT [8], future prequalification appears unlikely. However, given that ARCO^®^ is marketed in the private sector in a range of countries in Africa, Asia and Oceania, it is important for the cost-effectiveness implications of this form of ACT to be assessed.

In PNG, ARCO^®^ can be purchased as a single-dose therapy across the counter at an average cost of $1.84 per tablet, which is significantly more expensive than subsidized AL therapy ($0.19 per tablet) that is usually widely available through government hospitals and clinics [13, 15]. Even if AN were efficacious as single-dose therapy (as recommended by the manufacturer), its cost-effectiveness in comparison to a treatment course of AL would still be questionable. However, preliminary studies of the safety, efficacy and tolerability of AN in PNG children found unacceptable rates of treatment failure after a single dose [9, 10]. Dose-ranging studies identified that a minimum of two daily doses was required for adequate Day 28 and Day 42 ACPR, while a 3-day treatment course was considered the most appropriate given WHO recommendations [9].

For falciparum malaria in the present study, AN and AL had similar treatment efficacy (100 and 97.8% for AN and AL, respectively) but AL was significantly more cost-effective therapy with a mean cost of US$7.66 per patient (PP analysis) compared to US$17.28 per patient for AN therapy. This finding is consistent with other analyses of AL in a usual care setting. A cost-effectiveness study comparing DHA–PQP and AL for uncomplicated malaria in Tanzanian children completed in 2012 reported a mean cost of US$8.40 from the provider’s perspective for successful treatment of a clinical case of uncomplicated malaria [21]. Considering currency inflation over a 5-year period, the present findings are also consistent with previous reports of the cost-effectiveness of AL for treatment of uncomplicated falciparum and vivax malaria in Papua New Guinean children in 2008 ($6.97 per treatment success) [5]. A potential complication in determining cost-effectiveness analyses across different malaria-endemic regions is the relative lack of data from South-east Asia compared to the African continent where a number of different donation/subsidization schemes ensure availability to all in need [13]. The maximum manufacturer prices for AL for children with uncomplicated malaria were recently set by the Global Fund at US$0.43–$1.22 per treatment course [12, 13] but these data are predominantly from African countries and it is not known how many other malaria endemic nations received subsidized anti-malarials under the Affordable Medicines Facility malaria (AMFm) scheme.

A further consideration for countries such as PNG is that, despite a decline in the prevalence of falciparum malaria, the prevalence of *P. vivax* has increased by 13–36% [22–24] to the point where it has become the predominant *Plasmodium* species causing infection and illness in young PNG children [25]. Primaquine cannot be given for routine radical cure of *P. vivax* mainly because of the risk of haemolysis due to the relatively high prevalence of glucose-6-phosphatase deficiency in PNG. In this situation, selection of an ACT with a long half-life component is desirable so that there is an extended period of prophylaxis against relapses from hypnozoites during recovery [26]. In the present study, AL was efficacious in only 30% of children with vivax malaria compared with 100% after treatment with AN [11]. This rate of AL treatment failure is similar to that reported in other studies conducted in similar geo-epidemiological settings [27–29]. It is the most likely reason why the present analysis found AN to be similarly cost-effective to AL in *P. vivax* infections in contrast to the marked disparity in the case of *P. falciparum* infections. The excess cost per treatment success with AN was $14.83 for *P. vivax* versus $436.82 for *P. falciparum*.

There are population data from PNG detailed enough to provide estimates of the benefit of ACT regimens. In 2009, 1,431,395 suspected malaria cases were treated in PNG with a reported 604 malaria-attributable deaths. This represents 21.3% of the total PNG population assuming no multiple presentations. Furthermore, a study of febrile patients presenting at five sentinel health facilities across PNG in 2008 reported a malaria slide positivity rate ranging from 2.2 to 74.9% [30]. The clinical trial data, on which this analysis is based, showed that 79% of uncomplicated cases were due to *P. falciparum* only, 14% to *P. vivax* only, and 6% to mixed *P. falciparum* and *P. vivax* infections. Projecting the trial PP usual care cost-effectiveness analysis to the general PNG population of 7,154,870 in 2012, and assuming that all suspected cases were confirmed as a worst case scenario, 213,358 and 28,499 more cases of *P. vivax* and *P. falciparum*, respectively, could be treated successfully every year if AN were used instead of AL, costing an extra US$14,663,345 per year. However, these estimates may have to be revised as, since the trial was conducted in 2012, two rounds of countrywide distributions of long lasting insecticide treated nets have resulted in a 6.7% reduction in the overall prevalence of malaria [31, 32]. It was estimated that the cost per life year saved by using AN instead of AL to treat uncomplicated malaria to be $12,165 for girls and $12,469 for boys (discounted). This may not be cost-effective and affordable given that the gross national income *per capita* in PNG in 2012 was $1820 [33, 34], unless the cost of AN is reduced considerably.

The present study had limitations. The analysis did not consider treatment compliance. All treatments are given over 3 days but AL requires two doses per day and the medication should be taken with supplementary fat (usually milk or biscuits), aspects which might reduce compliance. Although all children allocated AL in the trial had a therapeutic plasma lumefantrine concentration on Day 7, consistent with all six doses having been administered successfully [11], a cost-effectiveness study of ACTs in sub-Saharan Africa estimated compliance with ACTs to be only 30–60% compared with 85–95% for sulfadoxine–pyrimethamine [35]. As conventionally recommended in cost-effectiveness analyses, estimates were based on the primary endpoints of the intervention trial. In the usual care situation in countries such as PNG, diagnosis and response to treatment are not underpinned by PCR. In addition, microscopy and/or rapid diagnostic tests (RDTs) may not be available or reliable in areas of PNG and in other resource-poor tropical countries. A national cross-sectional survey conducted prior to the implementation of the revised PNG national malaria treatment programme found that that RDTs or functional microscopy was utilized in only 15% of health facilities [36]. As a result, presumptive treatment based on presenting symptoms is most likely to occur, as shown in the same survey where 96.4% of fever presentations received anti-malarial treatment including 82% of patients who tested negative for malaria by either RDT or microscopy [36]. Recent PNG surveys assessing 88 health centres across PNG in 2012 showed a substantial increase in the use of RDTs and microscopy across all health facilities between 2010 and 2012 (16.2% vs. 68.3%) [37]. Although an economic analysis of the impact of these changes was beyond the scope of the present study, the data suggest that a progressive reduction in RDT costs in parallel with increased availability may improve diagnostic accuracy in countries such as PNG, and thus reduce inappropriate treatment.

Other potential limitations were that, while uncertainty has been accounted for in the outcomes of the trial via bootstrapping, the estimates of cost per life year are meant to be indicative and so a probabilistic model that would allow quantification of uncertainty around these estimates was not built. The current analysis also did not consider the cost implications of the mixed *Plasmodium* infections but these were a minor component of the burden of disease (6.5% of total cases). Post-treatment gametocyte carriage and consequent malaria transmission was also not factored into the analyses. Opportunity costs, such as the time taken off work by parents needing to look after their sick child including transportation to the clinic or hospital, have not been addressed. Treatment that accelerates recovery might allow parents to return to work more quickly. Finally, there are sometimes unpredictable shortages of government supplies of approved drugs such as AL in PNG which means that parents have to fill prescriptions at commercial pharmacies at increased cost.

## Conclusions

AL proved the most cost-effective ACT treatment for both infecting *Plasmodium* species in PNG but its low efficacy against vivax malaria remains a concern. While, for programmatic reasons, the use of a single first-line therapy that is effective against both *P. falciparum* and *P. vivax* infections is preferred in regions where both species are transmitted, the superiority of AN against vivax malaria [11, 26] means that this ACT medicine should be reconsidered if there is a reduction in price and/or it should achieve WHO prequalification status. Since the first intervention trial [5] and related cost-effective analysis [6], DHA–PQP has received approval for WHO prequalification and is, therefore, included on the list of anti-malarial drugs which are subsidized through the Global Fund and AMFm initiative [38]. Given its superior efficacy over AL for vivax infections and more recent evidence of continued high efficacy in falciparum malaria [39, 40], DHA–PQP may need to be changed from second- to first-line treatment in PNG if AL efficacy wanes as has happened in parts of South-east Asia [41]. In the meantime, the present cost-effectiveness data support AL over AN for first-line treatment of uncomplicated malaria in PNG and geo-epidemiologically similar areas in Oceania and South-east Asia.

## Abbreviations

ACPR: adequate parasitological and clinical response
AMF: Affordable Medicines Facility malaria
AL: artemether–lumefantrine
ACT: artemisinin-based combination therapy
AN: artemisinin–naphthoquine
DHA–PQP: dihydroartemisinin–piperaquine
CI: confidence intervals
ETF: early treatment failure
Hb: haemoglobin
ICER: incremental cost-effectiveness ratio
KPC: Kunming Pharmaceutical Corporation
LCF: late clinical failure
LPF: late parasitological failure
mITT: modified intention to treat
PNG: Papua New Guinea
PP: per protocol
PCR: polymerase chain reaction
RDT: rapid diagnostic test
WHO: World Health Organization
